# Acceptor Stem Differences Contribute to Species-Specific Use of Yeast and Human tRNA^Ser^

**DOI:** 10.3390/genes9120612

**Published:** 2018-12-07

**Authors:** Matthew D. Berg, Julie Genereaux, Yanrui Zhu, Safee Mian, Gregory B. Gloor, Christopher J. Brandl

**Affiliations:** Department of Biochemistry, The University of Western Ontario, London, ON N6A 5C1, Canada; jgenerea@uwo.ca (J.G.); yzhu633@uwo.ca (Y.Z.); mmian6@uwo.ca (S.M.); ggloor@uwo.ca (G.B.G.)

**Keywords:** tRNA^Ser^, mistranslation, anticodon, functional conservation

## Abstract

The molecular mechanisms of translation are highly conserved in all organisms indicative of a single evolutionary origin. This includes the molecular interactions of tRNAs with their cognate aminoacyl-tRNA synthetase, which must be precise to ensure the specificity of the process. For many tRNAs, the anticodon is a major component of the specificity. This is not the case for the aminoacylation of alanine and serine to their cognate tRNAs. Rather, aminoacylation relies on other features of the tRNA. For tRNA^Ser^, a key specificity feature is the variable arm, which is positioned between the anticodon arm and the T-arm. The variable arm is conserved from yeast to human. This work was initiated to determine if the structure/function of tRNA^Ser^ has been conserved from *Saccharomyces cerevisiae* to human. We did this by detecting mistranslation in yeast cells with tRNA^Ser^ derivatives having the UGA anticodon converted to UGG for proline. Despite being nearly identical in everything except the acceptor stem, human tRNA^Ser^ is less active than yeast tRNA^Ser^. A chimeric tRNA with the human acceptor stem and other sequences from the yeast molecule acts similarly to the human tRNA^Ser^. The 3:70 base pair in the acceptor stem (C:G in yeast and A:U in humans) is a prime determinant of the specificity. Consistent with the functional difference of yeast and human tRNA^Ser^ resulting from subtle changes in the specificity of their respective SerRS enzymes, the functionality of the human and chimeric tRNA^Ser^_UGG_ molecules was enhanced when human SerRS was introduced into yeast. Residues in motif 2 of the aminoacylation domain of SerRS likely participated in the species-specific differences. Trp290 in yeast SerRS (Arg313 in humans) found in motif 2 is proximal to base 70 in models of the tRNA-synthetase interaction. Altering this motif 2 sequence of hSerRS to the yeast sequence decreases the activity of the human enzyme with human tRNA^Ser^, supporting the coadaptation of motif 2 loop–acceptor stem interactions.

## 1. Introduction

A fundamental property of all cells is the conversion of DNA sequence into protein via an RNA intermediate. The genetic code, the mechanism of translation, and the machinery involved are highly conserved in all domains of life. Fidelity during translation is maintained at two steps. First, specific aminoacyl-tRNA synthetases (aaRS) are responsible for ligating the correct amino acid onto their cognate tRNAs. Secondly, decoding at the ribosome ensures the correct pairing between a tRNA anticodon and the mRNA codon. Errors at either step can result in the incorporation of the wrong amino acid into the growing polypeptide chain, known as mistranslation [1].

Each aaRS selectively recognizes its cognate tRNAs through individual bases and base pairs, known as identity elements. In two dimensions, the canonical tRNA structure consists of an acceptor stem, where the amino acid is ligated, and three stem-loop structures (reviewed in Reference [2]). From 5′ to 3′ these are the D-arm, the anticodon stem-loop that interacts with the mRNA at the ribosome, and the T-arm, which contributes to the interaction with elongation factor thermo unstable (EF-Tu) to recruit the tRNA to the ribosome. Serine, leucine, and bacterial tyrosine tRNAs have an addition arm, i.e., the variable arm, between the anticodon and T-arm. In three dimensions, all tRNAs fold into a L-shape (reviewed in Reference [3]). In this structure, the acceptor stem and T-arm interact to form one part of the L and the anticodon stem and D-arm interact to form the other. Many aaRS enzymes span the entire length of the tRNA with both the anticodon and acceptor stem being major determinants for recognition [4,5,6]. In contrast, the anticodon is not a determinant for seryl- and alanyl-tRNA synthetases [6]. For the alanyl-tRNA synthetase (AlaRS), the major identity element is a G3:U70 base pair in the acceptor stem [7,8,9,10]. A major determinant for seryl-tRNA synthetase (SerRS) is the variable arm [11,12,13].

All aaRSs catalyze the attachment of an amino acid onto the 3′ end of a tRNA in a two-step reaction. In the first step, an amino acid is recognized and activated with ATP to form an aminoacyl-adenylate. In the second step, the activated amino acid is transferred to the terminal 3′ adenosine of the tRNA (reviewed in Reference [14]). The aaRSs are divided into two classes based on their catalytic domains [14,15,16]. Class I aaRS enzymes contain a Rossmann fold with two consensus motifs (HIGH and KMSKS) that interact with ATP [15,17,18]. In class II enzymes, a unique seven sheet antiparallel beta fold [19] containing the highly degenerate motif 2 (FRXE) and motif 3 (GXGXGF[D/E]R) are responsible for binding ATP [15,20,21].

Class II enzymes are composed of a catalytic domain, often an editing domain and, in all members except AlaRS and SerRS, an anticodon binding domain [22]. Like other class II enzymes, SerRS approaches the tRNA acceptor stem from the major groove side [12,23]. In the catalytic site, beta sheets form the base of a pocket with two sides composed of large hairpins [22]. A conserved phenylalanine near the end of motif 2 stacks with the adenine ring of ATP, while a conserved arginine interacts with the alpha-phosphate [12]. The hairpin loop between two beta-strands of motif 2 contacts the upper bases of the acceptor stem [12,22,23,24,25]. The main recognition element of SerRS is the unique long variable arm, which is bound by an N-terminal tRNA binding domain comprising two alpha helices in all organisms except methanogens [12,26]. The enzyme functions as a homo-dimer, where the tRNA binding domain of one subunit binds the variable arm and positions the 3′ end of the tRNA in the active site of the other subunit [12,27,28]. Additional in vitro and in vivo experiments have identified other weak identity elements in the acceptor stem of bacterial tRNA^Ser^ [29,30].

Translation is a fundamental process where both the mechanism and the molecules involved in steps such as aminoacylation of tRNAs are conserved. This is evident from the ability of heterologous aaRS to complement deletions of native synthetase genes. For example, the human LysRS rescues a deletion of *Escherichia coli* LysRS [31], and the human AlaRS can replace the native yeast enzyme [32]. More recently, complementation of MetRS in yeast by the human enzyme was used to investigate disease-related variants [33]. However, coevolution of tRNA and synthetase pairs often results in decreased or abolished tRNA function when expressed in non-native organisms. For example, Burke et al. [34] found that human tRNA^Pro^ is not aminoacylated by *E. coli* ProRS due to differences in the acceptor stem and D-arm of the tRNA. Edwards et al. [35] used a tyrosine tRNA suppressor from *E. coli* to suppress stop codons in yeast and found it was not aminoacylated by yeast TyrRS but rather was aminoacylated with leucine. In addition, approaches to encode noncanonical amino acids at stop codons rely on orthogonal tRNA:aaRS pairs, often transplanted from other organisms, that do not interact with endogenous tRNAs or aaRSs (reviewed in Reference [36]). 

This work was initiated to determine if the structure/function of tRNA^Ser^ has been conserved from *Saccharomyces cerevisiae* to human. We analyzed human tRNA^Ser^ function in *S. cerevisiae* by detecting mistranslation and suppression with tRNA^Ser^ derivatives having the UGA anticodon converted to UGG for proline. We found that despite the conservation of tRNA^Ser^, differences in the acceptor stem result in reduced functionality of the human tRNA in yeast. The 3:70 base pair in the acceptor stem (C:G in yeast and A:U in humans) is a prime determinant of the specificity. Toxicity of the humanized tRNAs increases when human SerRS is introduced into yeast, suggesting a role for aminoacylation in the differential function. 

## 2. Materials and Methods

### 2.1. Yeast Strains

Yeast strains are derivatives of the wild-type haploid strain BY4742 (Table S1; Reference [37]). The *tti2* disruption strain complemented by *tti2-L187P* on a *LEU2* centromeric plasmid (CY7020) has been previously described [10]. The *met22Δ* strain (CY7640) was derived from a spore colony of the yeast magic marker strain in the BY4743 diploid background [38].

Yeast strains were grown in yeast peptone media containing 2% glucose or synthetic media supplemented with nitrogenous bases and amino acids at 30 °C. Selections for CY7020-derived strains were on yeast peptone dextrose (YPD) plus 5% (*v/v*) ethanol. Cells were grown in dropout medium to stationary phase, 10-fold serially diluted, and spotted onto the indicated medium.

Growth curves were generated by diluting saturated cultures to OD_600_ ~ 0.1 in minimal media and incubating at 30 °C. OD_600_ was measured every 15 min using a BioTek Epoch 2 (BioTek, Winooski, VT, USA) microplate spectrophotometer for 24 h. 

### 2.2. Yeast Transformation

Approximately 10^6^ cells were pelleted and washed twice in an equivalent volume of 100 mM lithium acetate, 1.0 mM ethylenediaminetetraacetic acid, and 10 mM TrisHCl (pH 7.5; LiAc) and suspended in 200 μl of LiAc. Five milligrams of denatured calf thymus DNA and ~1.0 μg of plasmid DNA was incubated with 100 μl of cells for 15 min at 30 °C. Then, 1.0 mL of 40% polyethylene glycol 4000 was added, and the cells were incubated 15 min at 30 °C. Dimethyl sulfoxide was added to 7%, and the cells were incubated at 42 °C for 15 min. Cells were pelleted, plated on selective medium, and grown at 30 °C.

### 2.3. Plasmid Constructs

*SUP17* [*Sc*tRNA^Ser^_UGA_] including ~300 bp upstream and downstream and either the wild-type anticodon (UGA; pCB3076) or the proline anticodon (UGG; pCB3082) in the *URA3* centromeric plasmid YCplac33 have been previously described [39]. The G26A derivative of pCB3082 (pCB4023) and A4G derivative of pCB3082 (pCB4097) were identified by selection [39]. DNA containing the sequence encoding human tRNA^Ser^ (chr6.trna172-SerUGA) with proline anticodon UGG inserted directly in place of *SUP17* (*Hs*tRNA^Ser^_UGG_; see Figure S1) was purchased from Invitrogen (Pleasanton, CA, USA). This was digested with *Pst*I and *EcoR*I and inserted into YCplac33 (pCB4062). The *SUP17-*human acceptor stem chimera (*ch*tRNA^Ser^_UGG_) with proline anticodon was constructed by two-step PCR using the outside primers UG5953 and UG5954, internal primers VD5993 and VD5994 to alter the 3’ end of the acceptor stem, VD5578 and VD5579 to alter the 5′ end of the acceptor stem, and pCB4062 as template. The molecule was cloned as a *Hind*III-*Eco*RI fragment into YCplac33 (pCB4122). (See Table S2 for oligonucleotides.) The A4G:U69C derivative of pCB3082 (pCB4145) was similarly constructed using pCB4097 as template and the inside primers VF8687 and VF8688. The A3C:T70G derivative of the chimeric tRNA^Ser^_UGG_ (pCB4336) was similarly constructed using outside primers UG5953 and UG5954, inside primers WE0866 and WE0867 to alter the 3’ end, WE0868 and WE0869 to alter the 5’ end, and pCB4122 as the template.

The cloned coding region of human seryl-tRNA synthetase (SARS) was purchased from NovoPro Biosciences Inc. (Cat# 715151-2, Shanghai, China; RefSeq NM_006513). This was amplified by PCR using oligonucleotides VD5232 and VD5803 and cloned as a *Not*I-*Eco*RI fragment into YCplac111 containing a *DED1* promoter and myc^9^-tag (pCB4329; Hoffman et al. [40]) and YEplac181 containing a *DED1* promoter and myc-tag (pCB4355). Note that codon 435, which codes for C in the purchased clone, was converted by two-step PCR to an R codon to resemble those more commonly found in mammalian SerRS. A derivative of SARS with mutations T312A and R313W was constructed by ligating an *Nco*I-*Eco*RI fragment synthesized by PCR with oligonucleotides WF1846 and VD5803 into pCB4355 to give pCB4357. *S. cerevisiae SES1* was similarly inserted into YCplac111 (pCB4342) and YCplac181 (pCB4356) after PCR with oligonucleotides WF1727 and UK0551.

The centromeric plasmid containing *HSE-eGFP* has previously been described [41] and was kindly provided by Martin Duennwald.

### 2.4. Western Blot Assay

Yeast extract prepared by grinding with glass beads [42] was separated by SDS-PAGE and transferred to PVDF membrane (Roche Applied Science, Penzberg, Germany). Anti-myc (9E10, Sigma-Aldrich, St. Louis, MO, USA) was used at a ratio of 1:5000. Secondary antibody (anti-Mouse IgG HRP, Promega, Madison, WI, USA) was used at a ratio of 1:10,000 and was detected using SuperSignal West Pico Chemiluminiscent Substrate (Thermo Scientific, Waltham, MA, USA). 

### 2.5. Fluorescence Heat Shock Reporter

Yeast strains containing the heat shock response element (HSE)-*eGFP* reporter were grown to stationary phase in a medium lacking leucine and uracil, diluted 1:50 in the same medium, and grown for 6 h at 30 °C. Cell densities were normalized to OD_600_ before measuring fluorescence. Fluorescence was measured with a BioTek Synergy H1 microplate reader at an emission wavelength of 528 nm using Gen5 2.08 software. The mean relative fluorescence units were calculated across three technical and three biological replicates for each strain.

### 2.6. Mutual Information Analysis

Coevolving residues within SerRS were identified following the protocol outlined by Dickson and Gloor [43]. Briefly, crystal structures of SerRS from *Candida albicans* (3QNE; [44]), *Aquifex aeolicus* (2DQ3), *Pyrococcus horikoshii* (2ZR2; [45]), *Homo sapiens* (4L87; [46]), *Naegleria fowleri* (6BLJ), and *Thermus thermophilus* (1SET; [47]) from the Protein Data Bank were used to construct a structural alignment. ASN.1 files for each structure were obtained from the Molecular Modeling Database, and the structures were aligned in Cn3D [48] to obtain block structures for sequence alignment. Seryl-tRNA synthetase sequences were obtained from the UniProt Reference Clusters 50 (UniRef50) dataset as FASTA files (Supplemental File S1), imported into Cn3D and block aligned with the SerRS structures. Sequences that were not annotated as cytoplasmic SerRS, as well as hypothetical proteins, were removed. Coevolution scores were calculated with MIpToolset [49]. 

## 3. Results and Discussion

**Figure 1:**
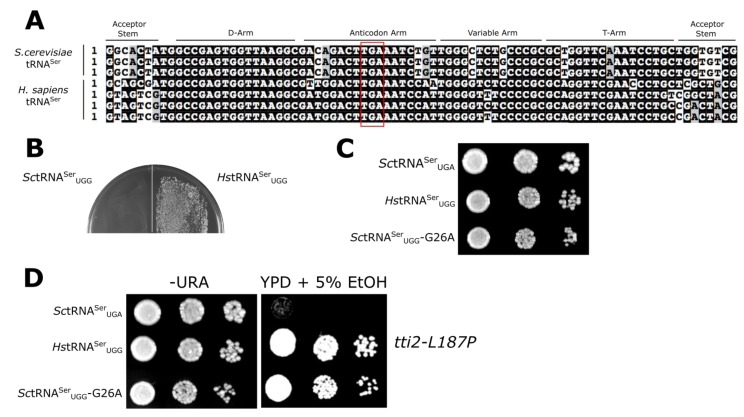
Human (*Homo sapiens*) tRNA^Ser^_UGA_ functions inefficiently in *Saccharomyces cerevisiae*. (**A**) Sequence alignment of genes encoding tRNA^Ser^_UGA_ isodecoders from *S. cerevisiae* and humans. The red box highlights the anticodon. Sequences were obtained from the GtRNAdb ([52]; http://gtrnadb.ucsc.edu/index.html) and aligned with MUSCLE (https://www.ebi.ac.uk/Tools/msa/muscle/). (**B**) *URA3* centromeric plasmid (YCplac33) expressing *S. cerevisiae* (*SUP17*) or human (tRNA-Ser-TGA-3-1) tRNA^Ser^ genes with their UGA anticodon converted to UGG for proline [*Sc*tRNA^Ser^_UGG_ and *Hs*tRNA^Ser^_UGG_] were transformed into the wild-type yeast strain BY4742 using a lithium acetate protocol and grown for 3 days at 30 °C. (**C**) The growth of BY4742 containing a centromeric plasmid expressing *Hs*tRNA^Ser^_UGG_ was compared to strains containing wild-type *Sc*tRNA^Ser^_UGA_ and the mistranslating *Sc*tRNA^Ser^_UGG_-G26A. Strains were grown in medium lacking uracil for 40 h, then 10-fold serially diluted, spotted on minimal plates lacking uracil, and grown for 2 days at 30 °C. (**D**) Plasmids expressing *Sc*tRNA^Ser^_UGA_, *Hs*tRNA^Ser^_UGG_, or *Sc*tRNA^Ser^_UGG_-G26A were transformed into the *tti2-L187P* strain CY7020, streaked for individual colonies, grown in minimal medium lacking uracil to stationary phase, then 10-fold serially diluted, spotted on minimal plates lacking uracil (-URA) or yeast peptone dextrose (YPD) containing 5% ethanol, and grown at 30 °C for 2 days (for -URA) or 3 days (for YPD + 5% ethanol).

The processes involved in translation are conserved across all species. Not surprisingly, there is significant similarity in the key molecules involved [50,51]. In this work, our goal was to investigate the cross-species similarities and differences in tRNA function by analyzing the activity of human tRNA^Ser^ in *S. cerevisiae.* To begin the analysis, we aligned the tRNA^Ser^_UGA_ isodecoders from *S. cerevisiae* and human (Figure 1A). Of the 83 nucleotides, 60 are conserved in both sets of tRNAs. Fifteen positions are always different between the three identical yeast tRNAs and the four human tRNAs. The variable arms are conserved in length and G:C content.

### 3.1. Mistranslation Assay for tRNA^Ser^ Function

We previously identified a stress-sensitive allele (L187P) of the co-chaperone protein *TTI2* that is suppressed by mistranslation of L187P with serine [39]. A gene expressing yeast *SUP17* (*Sc*tRNA^Ser^) with the UGA anticodon of the tRNA changed to UGG (to decode proline codons) cannot be introduced into yeast [39] due to high levels of mistranslation of serine at proline codons. Dampening the function of the tRNA with secondary mutations allows the introduction of tRNA^Ser^_UGG_ variants. If the tRNA^Ser^_UGG_ retains function, it is aminoacylated with serine and inserts (mistranslates) serine for proline at a level sufficient to suppress *tti2-L187P*. If the tRNA^Ser^_UGG_ variant is not functional, it can be introduced into yeast but does not mistranslate and thus does not suppress *tti2-L187P*. In our assay tRNA^Ser^_UGG_ competes with endogenous tRNA^Pro^ for the decoding of *tti2-L187P* mutation. We used this system to analyze the activity of human tRNA^Ser^_UGA_ in *S. cerevisiae* after converting the anticodon to UGG. A gene encoding human tRNA^Ser^ (chr2.trna21) with the UGA anticodon converted to UGG was inserted into the *SUP17* locus with ~300 bp of 5′ and 3′ flanking yeast sequence and cloned into the yeast *URA3* centromeric plasmid YCplac33 (see Figure S1 for the gene sequence). As shown in Figure 1B, the human gene expressing tRNA^Ser^_UGG_ (*Hs*tRNA^Ser^_UGG_) could be transformed into the wild-type strain BY4742, in contrast to *Sc*tRNA^Ser^_UGG_ [39]. The growth of this strain on a minimal plate was slightly slower than a strain containing YCplac33 alone and slightly faster than a strain containing *Sc*tRNA^Ser^_UGG_ with a G26A mutation (Figure 1C; see Reference [39]). The reduced toxicity of the human tRNA^Ser^_UGG_ indicates that its activity in yeast is diminished relative to *S. cerevisiae* tRNA^Ser^_UGG_. To determine if the human tRNA^Ser^ functions in yeast, *Hs*tRNA^Ser^_UGG_ was transformed into CY7020, which contains the crippled *tti2-L187P* allele. As shown by growth on medium containing 5% ethanol (Figure 1D), *Hs*tRNA^Ser^_UGG_ suppressed the stress sensitive growth caused by *tti2-L187P*. The human tRNA thus has partial function in yeast. 

**Figure 2:**
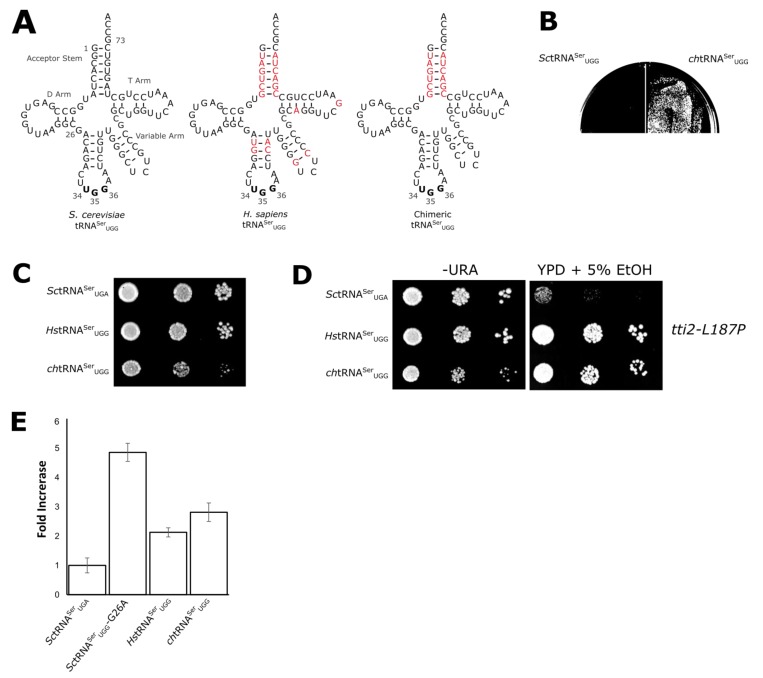
The human tRNA^Ser^ acceptor stem decreases the functionality of yeast tRNA^Ser^. (**A**) Secondary structures of tRNA^Ser^_UGG_ derivatives used in these studies. The chimeric molecule contains the human acceptor stem on the yeast tRNA. Differences with the *S. cerevisiae* tRNA^Ser^_UGG_ (*SUP17_UGG_*) are in red. (**B**) *URA3* centromeric plasmids expressing the chimeric tRNA (*ch*tRNA^Ser^_UGG_) with a UGG anticodon or *Sc*tRNA^Ser^ (*SUP17*) with a UGG anticodon were transformed into BY4742 and grown at 30 °C for 2 days on a minimal plate. (**C**) BY4742 expressing *Sc*tRNA^Ser^_UGA_, *Hs*tRNA^Ser^_UGG_, or *ch*tRNA^Ser^_UGG_ were grown overnight in minimal media, serially diluted, plated on media lacking uracil, and grown at 30 °C for 2 days. (**D**) Plasmids expressing *Sc*tRNA^Ser^_UGA_, *Hs*tRNA^Ser^_UGG_, or *ch*tRNA^Ser^_UGG_ were transformed into the *tti2-L187P* strain CY7020, streaked for individual colonies, grown in minimal medium for 40 h, 10-fold serially diluted, spotted on minimal plates lacking uracil (-URA) or yeast peptone dextrose (YPD) containing 5% ethanol, and grown at 30 °C for 2 days (for -URA) or 3 days (for YPD + 5% ethanol). (**E**) A reporter plasmid containing GFP expressed from a Hsf1-activated promoter was transformed into BY4742 also containing centromeric plasmids expressing wild-type *Sct*RNA^Ser^_UGA_, *Hs*tRNA^Ser^_UGG_, or *ch*tRNA^Ser^_UGG_. Starter cultures were grown in minimal medium, diluted into fresh selective medium, and grown for eight hours. Induction of the Hsf1-GFP was measured by fluorescence at 528 nm. Numbers shown are the average of five biological replicates performed in duplicate with the standard deviation shown.

Because 7 of the 15 differences always found between yeast and human tRNA^Ser^ are in the acceptor stem (Figure 1A), we engineered a chimeric tRNA with the human acceptor stem substituted on the *S. cerevisiae* tRNA and with a UGG anticodon (*ch*tRNA^Ser^_UGG_; Figure 2A). The chimeric tRNA^Ser^_UGG_ mimicked *Hs*tRNA^Ser^_UGG_ in its ability to be transformed into BY4742 (Figure 2B). As shown in Figure 2C, *ch*tRNA^Ser^_UGG_ was more toxic than *Hs*tRNA^Ser^_UGG_, suggesting that at least one other difference between *S. cerevisiae* and human tRNA^Ser^ exists outside the acceptor stem (discussed in Section 3.4). The chimeric tRNA was also functional as determined by its ability to suppress *tti2-L187P* when transformed into CY7020 (Figure 2D). 

The mistranslation experiments we performed were a balance between improved growth due to suppression of *tti2-L187P* and toxicity due to proteome-wide mistranslation. To provide another indication of the toxicity arising from mistranslation due to each of the tRNAs, we measured heat shock induction in the strains using a reporter plasmid containing GFP expressed from an Hsf1-activated promoter (Figure 2E). *Hs*tRNA^Ser^_UGG_ and *ch*tRNA^Ser^_UGG_ resulted in a heat shock response 2.1- and 2.8-fold, respectively, greater than the control. *Sc*tRNA^Ser^_UGG_-G26A resulted in a 4.8-fold increase in the heat shock response. These results suggest that the toxicity of human and chimeric tRNAs is the result of a loss of proteostasis due to mistranslation.

### 3.2. Evaluating Mechanisms for the Reduced Functionality of Human tRNA^Ser^

**Figure 3:**
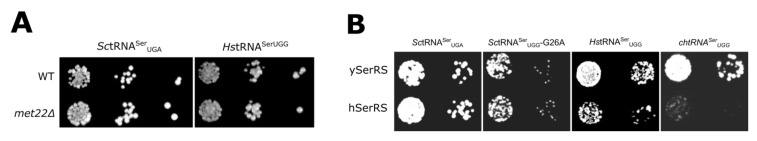
Evaluating mechanisms of decreased function of human tRNA^Ser^_UGG_ in yeast. (**A**) BY4742 (*MET22*) and CY7641 (*met22Δ*) containing either wild-type (WT) *Sc*tRNA^Ser^_UGA_ or *Hs*tRNA^Ser^_UGG_ were grown to stationary phase in media lacking uracil before cells were spotted in 10-fold serial dilutions onto a plate lacking uracil and grown at 30°. (**B**) Expression of human SerRS, SARS, increases the functionality and thus the toxicity of HstRNA^Ser^_UGG_ and *ch*tRNA^Ser^_UGG_ in yeast. Yeast strain BY4742 containing a *URA3* centromeric plasmid expressing *Sc*tRNA^Ser^_UGA_, *Sc*tRNA^Ser^_UGG_-G26A. *Hs*tRNA^Ser^_UGG_ or *ch*tRNA^Ser^_UGG_ and the *LEU2* multicopy plasmid YCplac181 containing myc-tagged yeast *SES1* (ySerRS) or human SARS (hSerRS) were grown to stationary phase in media lacking uracil and leucine. Cell densities were normalized, and cultures were spotted in 10-fold serial dilutions on the same media.

Differences between yeast and human tRNA^Ser^ could be due to any step leading to the functionality of the tRNA. Included in this are factors that contribute to the cellular concentration of the tRNA. Although it is difficult to compare the levels of the mistranslating tRNAs because the genomically encoded copies of yeast tRNA^Ser^_UGA_ differ from *Sc*tRNA^Ser^_UGG_ by only one nucleotide, we addressed a possible role for the rapid tRNA decay (RTD) pathway in the function of *Hs*tRNA^Ser^_UGG_. *Hs*tRNA^Ser^_UGG_ was transformed into a *met22Δ* strain where the RTD pathway is inhibited [53,54]. If the mistranslating human tRNA was being turned over by the RTD pathway, we would expect it to be more toxic in a *met22Δ* strain, where it would accumulate at higher levels and result in increased levels of mistranslation. The toxicity of *Hs*tRNA^Ser^_UGG_ was not increased in the *met22Δ* strain (Figure 3A), suggesting the human tRNA is not being turned over by the RTD pathway.

If the functionality and resulting mistranslation of *ch*tRNA^Ser^_UGG_ was reduced relative to *Sc*tRNA^Ser^ because of decreased aminoacylation, we would expect that toxicity would increase if human SerRS, SARS, was also introduced into yeast cells. We amplified the coding region of SARS by PCR and cloned it 3’ of the yeast *DED1* promoter and a myc tag. The myc-tagged SARS is expressed in *S. cerevisiae* strain BY4742 at a level comparable to *S. cerevisiae* Ses1 (Figure S2). Centromeric plasmids expressing *Sc*tRNA^Ser^_UGA_, *Sc*tRNA^Ser^_UGG_-G26A, *Hs*tRNA^Ser^_UGG_, and *ch*tRNA^Ser^_UGG_ were then transformed into strains expressing SARS and Ses1 on 2μ plasmids. Their growth was compared on minimal media lacking uracil and leucine. As shown in Figure 3B, the presence of human SARS reduces the growth of the strain containing either *Hs*tRNA^Ser^_UGG_ or *ch*tRNA^Ser^_UGG_ but not the strain containing *Sc*tRNA^Ser^_UGG_-G26A. The increased toxicity of *Hs*tRNA^Ser^_UGG_ and *ch*tRNA^Ser^_UGG_ in the presence of SARS further indicates that the acceptor stem contributes to differences in the aminoacylation of human and yeast tRNA^Ser^.

### 3.3. The tRNA^Ser^ 3:70 Base Pair Contributes to Species Specificity

**Figure 4:**
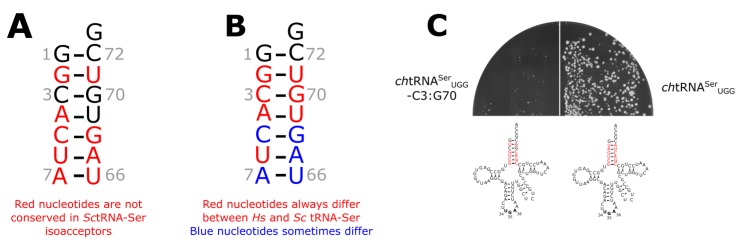
The acceptor stem 3:70 base pair is a determinant of yeast versus human tRNA^Ser^ specificity. (**A**) Variation in the acceptor stem of the *S. cerevisiae* tRNA^Ser^ isoacceptors. The acceptor stem of the tRNA encoded by *SUP17* is shown with the positions that vary in the different cytoplasmic tRNA^Ser^ isoacceptors colored in red. (**B**) Variation in the acceptor stems of tRNA^Ser^_UGA_ between *S. cerevisiae* and humans. The acceptor stem of the tRNA encoded by *SUP17* is shown with the bases always different between yeast and human colored in red and those sometimes-different colored in blue. (**C**) BY4742 expressing *ch*tRNA^Ser^_UGG_ or *ch*tRNA^Ser^_UGG_-C3:G70 on a *URA3* centromeric plasmid were transformed into BY4742 and grown at 30 °C for 2 days.

To begin to map the key bases within the acceptor stem that could account for the functional difference of human tRNA^Ser^ in yeast, we first compared sequences of the *S. cerevisiae* tRNA^Ser^ isoacceptors. We hypothesized that key bases for yeast SerRS should be conserved. Five bases in the acceptor stem are conserved in all yeast serine tRNAs. These are the G1:C72 and C3:G70 bases pairs and U69 (Figure 4A; see Figure S3 for the sequence of the tRNAs). Figure 4B shows the bases that differ between yeast and human tRNA^Ser^_UGA_ isodecoders. Only G1:C72 is conserved for all these tRNAs (see sequences in Figure 1A). U69 and the 3:70 base pair are thus strong candidates for a difference determinant between *S. cerevisiae* and human tRNA^Ser^. We evaluated the importance of U69 with a variant of *Sc*tRNA^Ser^_UGG_ containing the human G4:C69 base pair. A gene expressing this variant could not be transformed into yeast (Figure S4), indicating that it is fully functional and suggesting that U69 does not signal the specificity difference between yeast and human tRNA^Ser^. To test the 3:70 base pair, we constructed a variant of *ch*tRNA^Ser^_UGG_ with the yeast C3:G70 base pair (*ch*tRNA^Ser^_UGG_-C3:G70). Unlike the unmodified chimeric gene, *ch*tRNA^Ser^_UGG_ = C3:G70 gave rise to extremely slow growing colonies when transformed into BY4742 (Figure 4C). The C3:G70 mutation thus increases the functionality and toxicity of the chimeric tRNA in *S. cerevisiae*.

**Figure 5:**
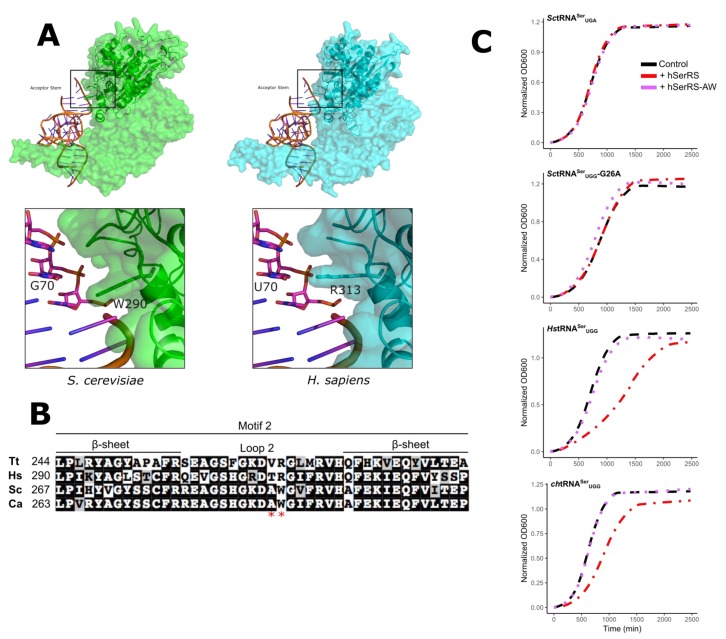
Sequence differences in Motif 2 of SerRS. (**A**) Model of motif 2 SerRS:tRNA^Ser^ interactions. The yeast (green) and human (cyan) SerRS were modeled using SWISS-MODEL [55] onto the *Thermus thermophilus* synthetase:tRNA co-crystal structure (PDB: 1SER; [12]). The region around motif 2 is boxed and expanded in the lower images. (**B**) Sequence alignment of SerRS motif 2 sequences from *T. thermophilus* (WP_024119136), *H. sapiens* (NP_006504), *S. cerevisiae* (NP_010306), and *Candida albicans* (XP_719967) using the default settings of MUSCLE (https://www.ebi.ac.uk/Tools/msa/muscle/). The red stars mark the residues mutated to the corresponding yeast residues. (**C**) Yeast strain BY4742 containing a *URA3* centromeric plasmid expressing *Sc*tRNA^Ser^_UGA_, *Hs*tRNA^Ser^_UGG_, *ch*tRNA^Ser^_UGG_. or *Sc*tRNA^Ser^_UGG_-G26A and the *LEU2* multicopy plasmid YEplac181 containing myc-tagged ySerRS (black), hSerRS (red), or mutant hSerRS-AW (purple) were grown to stationary phase in media lacking uracil and leucine, diluted to an OD_600_ ~ 0.1, and grown for 40 h at 30 °C. OD_600_ was measured every 15 min to generate growth curves.

The aminoacylation domain of the class II aaRS enzymes contains antiparallel β sheets. The loop between strands 2 and 3 comprise the conserved motif 2, which interacts with the tRNA acceptor arm [34]. The crystal structures of SerRS from several organisms have been solved. Included in these are the human enzyme and a structure of the *T. thermophilus* enzyme in complex with tRNA^Ser^. Unfortunately, in the latter, the acceptor stem is not fully resolved. Models for the interaction of SerRS with tRNA^Ser^ position motif 2 close to base pairs 3:70 and 4:69 of the acceptor stem (for example see Figure 5A and Reference [25]). To begin to identify what may account for the different acceptor stem preferences of the SerRS enzymes, we performed a sequence alignment of motif 2 from the human, *T. thermophilus*, *C. albicans*, and *S. cerevisiae* enzymes. The human and *T. thermophilus* enzymes prefer a A3:U70 base pair in the acceptor stem, whereas the two yeast enzymes prefer C3:G70. Two residues in motif 2 are conserved in the yeast enzymes that differ in the human and *T. thermophilus* enzymes (Figure 5B). Trp290 and Ala297 of the *S. cerevisiae* enzyme align with Arg and Gln, respectively, in *T. thermophilus* and human enzymes. Ala297 is not well conserved across yeast and fungal species containing C3:G70 tRNA^Ser^ (Figure S5); in contrast, the 290 position is conserved in yeast and is proximal to the base at position 70 (Figure 5A). We constructed a version of hSerRS, where residues T312 and R313 in motif 2 were converted to the corresponding yeast residues A and W. The mutated hSerRS was introduced into BY4742 containing wild-type *Sc*tRNA^Ser^_UGA_, *Sc*tRNA^Ser^_UGG_-G26A, *Hs*tRNA^Ser^_UGG_, or *ch*tRNA^Ser^_UGG_ (Figure 5C). The wild-type human SerRS decreased the growth rate in strains containing the human or chimeric tRNA^Ser^ but did not affect the strains containing the yeast tRNAs. In contrast, the hSerRS TR to AW mutant did not alter the growth rate in these strains when compared to the yeast SerRS, suggesting that T312 and R313 are important for the function of the human SerRS on the human tRNA.

Though it is difficult to exclude contributions from all other mechanisms, such as the exact cellular levels of each tRNA and their interactions, we suggest that aminoacylation is a component of the different functionality of human tRNA^Ser^ in *S. cerevisiae*. For class II enzymes, motif 2 contacts the acceptor stem [23,56,57,58]. Interactions between SerRS and tRNA^Ser^ suggest that these interactions influence aminoacylation by *S. cerevisiae* SerRS [12,25]. In addition, the toxicity, and thus the inferred functionality, of both *Hs*tRNA^Ser^_UGG_ and *ch*tRNA^Ser^_UGG_ was enhanced when human SerRS was introduced into yeast. Furthermore, altering the motif 2 sequence of hSerRS to resemble the yeast sequence decreases the activity of the human enzyme with human tRNA^Ser^. We do note that in our analysis, we compared one yeast serine isodecoders with one version of the same human isodecoders. Sequence differences in isodecoders families may result in different cross-species functionality. For tRNA^Ser^, the major identity element is the variable arm, both its length and its sequence [6,13,59]. Our data support the argument that the acceptor stem of tRNA^Ser^ also has a role in its aminoacylation and that these changes are species-specific. The role of the acceptor stem of tRNA^Ser^ parallels that seen for many other aaRS enzymes [6]. Interestingly, Shiba et al. [31] observed that the positioning of identity elements for specific tRNA isoacceptors is generally common across species, but the exact nature of these sequences is often species-specific. 

The possibility of a shared importance of the 3:70 base pair for aminoacylation by the class II enzymes AlaRS and SerRS is noteworthy. G3:U70 is unique for tRNA^Ala^ and highly conserved. The significance of the G3:U70 identity element for AlaRS has likely led to its parallel exclusion from other tRNAs. In fact, G3:C70 and A3:U70 show a somewhat limited distribution in non-alanine tRNAs in eukaryotic species (Figure S6), likely because of the mistranslation that would result from a single base mutation generating a G3:U70. These sequence limitations may have led to the 3:70 base pair’s role in recognition arising in some other tRNAs in some species. For many yeast and fungal species, the C3:G70 base pair is conserved in tRNA^Ser^-Ser. It is also conserved in *T. thermophilus* as A:U in all tRNA isoacceptors, which is interesting because of all the tRNAs, the four serine tRNAs are the only ones where A3:U70 is found. 

### 3.4. Unique Aspects of the SerRS Motif 2

It is interesting that the loop sequence in motif 2 in SerRS is distinct from other aaRS (Figure S7). The placement of Trp in the motif 2 loop of yeast SerRS was previously observed by Lenhard et al. [60]. They demonstrated that mutations to the loop region affect functionality. Our model proposes that Trp290 of yeast SerRS is a discriminating factor in the sequence preferences of the enzyme for the tRNA. Arginine in motif 2 of SerRS is frequently found with tRNA^Ser^ containing the A3:U70 base pair and in species containing a broader range of acceptor stem sequences, including humans, whereas tryptophan is frequently found where C3:G70 is the sole or predominant sequence as seen in yeast and fungi. Eichert et al. [25] examined the structure of minihelices with the *T. thermophilus* SerRS enzyme. They found that the side chain of Arg267 of the *T. thermophilus* enzyme (the equivalent of Trp290 in *S. cerevisiae* Ses1) interacts with the phosphate backbone of bases C69 and U70 via a water network. The exact positioning of Trp290 in the *C. albicans* structure is difficult to predict because of low electron density in this region; however, the hydrophobic Trp290 side chain almost certainly will have different interactions than a polar arginine. Tryptophan is infrequently found in base interactions [61], but hydrophobic residues occur in nucleic acid interactions through base stacking. For example, a tryptophan in the Tn5 transpose base stacks with a thymine of its substrate DNA [62]. This interaction requires distortion of the DNA duplex. Similar hydrophobic interactions occurring after base flipping are observed for other enzymes, including methyltransferases and endonucleases [63]. Further experiments will be required to determine if correct positioning of the tRNA acceptor stem of the Trp290 enzymes involves distortion of the base pairing. Alternatively, the Trp290 containing enzymes may compensate for the lack of interaction through additional contacts. To address this possibility, we analyzed the mutual information between each pair of positions within the aminoacylation domain of the cytoplasmic SerRS family of proteins to determine residues coevolving with Trp290 (Figure S8; Supplemental File S2). No residue was found to specifically coevolve with Trp290. We do note that although the role of the distinct motif 2 sequence found in SerRS may be in tRNA recognition, motif 2 more generally plays a role in ATP binding. This function could be indirectly affected by our mutations. 

The chimeric *S. cerevisiae* tRNA^Ser^ with the human acceptor stem was somewhat more toxic and induced a slightly greater heat shock response in yeast than the human tRNA^Ser^. This suggests that some of the other base differences between yeast and human tRNA^Ser^ molecules contribute a partial effect to tRNA function. These may include the differences in the anticodon stem, specifically at position 29 (A in *S. cerevisiae*, G in humans); through random selection, we have identified a G mutation in the yeast tRNA as having reduced function [64]. It is unlikely that this mutation affects aminoacylation given the lack of involvement of the anticodon stem-loop in the reaction. There are also differences in the T-arms of yeast and human tRNAs, which may influence interactions of the tRNA with the elongation factor EF-1α.

### 3.5. Applications for Mistranslation

tRNAs that mistranslate have roles in synthetic biology and potentially therapeutically. The latter arises because most diseases are the result of missense mutations that could be corrected through mistranslation. The synthetic biology applications include expanding the diversity of expressed products (statistical proteins) and controlling expression while transferring DNA between species. All these cases require tRNAs that mistranslate at a level below a toxic threshold. Our results indicate that a simple approach to achieve this is to use the inherent differences in function seen in the tRNAs from different species.

Translation requires multiple independent yet connected processes. Overall, these are highly conserved across species. It is, however, the differences that allow the enzymes involved in translation to be the targets of antibiotic and antimicrobial agents. The subtle differences seen for SerRS and the species specificity of its interactions with tRNA^Ser^ suggest its potential as a therapeutic target. 

## Supplementary Materials

The following are available online at http://www.mdpi.com/2073-4425/9/12/612/s1. Figure S1: Human tRNA^Ser^ sequence, Figure S2: Expression of human SerRS in yeast, Figure S3: Gene sequence of *S. cerevisiae* tRNA^Ser^ isoacceptors for cytoplasmic translation, Figure S4: Human tRNA^Ser^_UGG_ with G4:C69 is toxic in *S. cerevisiae*, Figure S5: Alignment of cytoplasmic SerRS motif 2 sequences from yeast and fungal species, Figure S6: Percent distribution of the base pair at 3:70 in the tRNAs in *Saccharomyces cerevisiae, Homo sapiens, Candida albicans, Thermus thermophilus* and *Escherichia coli*, Figure S7: Motif 2 sequences from the yeast class II aaRS enzymes Hts1, Figure S8: Co-evolution of cytoplasmic SerRS, Table S1: Yeast strains used in this study, Table S2: Oligonucleotides used in this study, File S1: SerRS Coevolution Sequences, File S2: SerRS Coevolution Mutual Information Output.
