# Supplementary material for: Acceptor Stem Differences Contribute to Species-Specific Use of Yeast and Human tRNASer
Source: Genes (Basel). 2018 Dec 7;9(12):612. doi: 10.3390/genes9120612 (PMC6316282; doi:10.3390/genes9120612)
Supplement: Supplementary file 1 [file genes-09-00612-s001.zip › Berg et al. - Supplemental Data.docx]

**Supplemental Material**

**Table S1. Yeast strains used in this study**

| Strain | Genotype/Parent | Plasmid(s) | Reference |
| --- | --- | --- | --- |
| BY4742 | *MATα his3∆0 leu2∆0 lys2∆0 ura3∆0* |  | Winzeler and Davis, 1997 |
| CY7020 | *MATα his3Δ1 leu2Δ0*  *lys2Δ0 met15Δ0*  *ura3Δ0 tti2Δ-met5Δ::Tn10luk* | YCplac111-*DED1-tti2_L187P_* | Hoffman *et al.,* 2016 |
| CY7640 | *MATα his3Δ1 leu2Δ0 ura3Δ0 met22::kanMX* |  | Tong *et al.,* 2001 |

**Table S2. Oligonucleotides used in this study**

| **Name** | **Sequence** | **Description** |
| --- | --- | --- |
| UG5953 | TCTAAGCTTCGGACGATTGCCAACCGCCGAA | *SUP17* |
| UG5954 | CTGCAGAATTCCGCGGAAATTAGCACGGCC | *SUP17* |
| WE0866 | attgttttagtCgtcgtggccgagtggtt | *ch*tRNA^Ser^_UGG_ A3C |
| WE0867 | ggccacgacGactaaaacaatgtaggttattt | *ch*tRNA^Ser^_UGG_ A3C |
| WE0868 | aatcctgccgacGacgttatttttttatttttattt | *ch*tRNA^Ser^_UGG_ U70G |
| WE0869 | taaaaaaataacgtCgtcggcaggatttgaaccag | *ch*tRNA^Ser^_UGG_ U70G |
| VD5232 | aaataagcggccgcgatggtgctggatctggatt | Human SerRS (SARS) |
| VD5803 | ccatccgaattcaagcatcggtgacctccatgt | Human SerRS (SARS) |
| WF1846 | tcCCATGGCCGTGACGCCUGGGGCATCTTCCGAGTC | SARS TR-AW |
| WF1727 | TTTGCGGCCGCGTTGGACATCAACCAATTTA | Ses1 |
| UK0551 | TCTAGAGAGCTCTATTATGCAACCATGTATATAAGATTATGGA | Ses1 |
| VD5578 | attgttttagtagtagtcgtggccgagtggttaaggc | 5’ *ch*tRNA^Ser^_UGG_ |
| VD5579 | ctcggccacgactactaaaacaatgtaggttatttaag | 5’ *ch*tRNA^Ser^_UGG_ |
| VD5993 | caaatcctgccgactacgttatttttttatttttatttttt | 3’ *ch*tRNA^Ser^_UGG_ |
| VD5994 | taaaaaaataacgtagtcggcaggatttgaaccagcg | 3’ *ch*tRNA^Ser^_UGG_ |
| VF8687 | aaatcctgctggcgtcgttatttttttatttttat | *Sc*tRNA^Ser^_UGG_ U69C |
| VF8688 | aaataacgacgccagcaggatttgaaccagcg | *Sc*tRNA^Ser^_UGG_ U69C |

CTGCAGCCGATGCTTATATACGGACGATTGCCAACCGCCGAAAAGGTTCAAGCCAAGAACAAAAAGTAGAGAGAATACCCCAACAATAGCATTTGATGGCTAAACGGCAGTGCCCGTTAATACTATTCAAATTATACCCCGCATTAATAATTCCTGTCCCTTTAGTCTCGTCCTTCAGCCGTATAGCCGCAAAACTTCGTTCAATGATTTCATGCCATCATTACACCGCGAGTCGTGGGTGCATTGTAGTTTATTACGAAAAGTATCTACAATACTTGCTTAAATAACCTACATTGTTTTAGTAGTCGTGGCCGAGTGGTTAAGGCGATGGACTTGGAATCCATTGGGGTCTCCCCGCGCAGGTTCGAATCCTGCCGACTACGTTATTTTTTTATTTTTATTTTTTTTTTATCGCTTACTGATTATCAGATATCTTCGACAGCCTCACACAGATTGGTGATCACGCACCCATAATCATTTCTTCGGGCATGCTCCTATTAGCCACGGTTCGCAGAATAATCTGCGCGATGTTATTCACCAAGACCGTGGAGTCTCCTTTTCGGTGGGATCCGCTTATATCCGTATGCGTCGGTTGCTTTCTCGGGGAAAGGAAAAGGAGAAAGCCCGGATAACACGACACAAGAGTCATTCGTTATGCACGGACACAGGGAATCGTGCGGAGCCGGGGATAAGAGGCCGTGCTAATTTCCGCGGAAttc

**Figure S1.** Human tRNA^Ser^ (chr6.trna172-SerUGA) with a UGG anticodon in the SUP17 locus. The tRNA coding region is underlined.

*
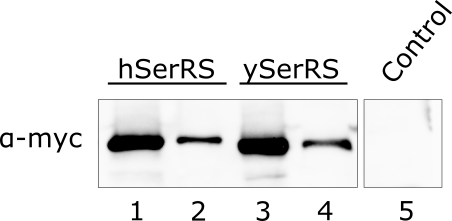
*

**Figure S2.** Expression of human SerRS in yeast. BY4742 was transformed with a URA3 centromeric plasmid (YCplac33) expressing either myc-tagged hSerRS (*SARS1*) or myc-tagged ySerRS (*SES1*) from the *DED1* promoter or with YCplac33 (Control). Transformed cells were grown in minimal medium and protein isolated by bead lysis. 50 μg of protein was separated by SDS-PAGE and Western blotted with anti-myc antibody.

>chrIV.trna14-AGA

GGCAACTTGGCCGAGTGGTTAAGGCGAAAGATTAGAAATCTTTTGGGCTTTGGCCCGCGCAGGTTCGAGTCCTGCAGTTGTCG

>chrI.trna4-AGA

GGCAACTTGGCCGAGTGGTTAAGGCGAAAGATTAGAAATCTTTTGGGCTTTGCCCGCGCAGGTTCGAGTCCTGCAGTTGTCG

>chrII.trna4-AGA

GGCAACTTGGCCGAGTGGTTAAGGCGAAAGATTAGAAATCTTTTGGGCTTTGCCCGCGCAGGTTCGAGTCCTGCAGTTGTCG

>chrIV.trna22-AGA

GGCAACTTGGCCGAGTGGTTAAGGCGAAAGATTAGAAATCTTTTGGGCTTTGCCCGCGCAGGTTCGAGTCCTGCAGTTGTCG

>chrIV.trna4-AGA

GGCAACTTGGCCGAGTGGTTAAGGCGAAAGATTAGAAATCTTTTGGGCTTTGCCCGCGCAGGTTCGAGTCCTGCAGTTGTCG

>chrV.trna1-AGA

GGCAACTTGGCCGAGTGGTTAAGGCGAAAGATTAGAAATCTTTTGGGCTTTGCCCGCGCAGGTTCGAGTCCTGCAGTTGTCG

>chrVII.trna28-AGA

GGCAACTTGGCCGAGTGGTTAAGGCGAAAGATTAGAAATCTTTTGGGCTTTGCCCGCGCAGGTTCGAGTCCTGCAGTTGTCG

>chrVIII.trna9-AGA

GGCAACTTGGCCGAGTGGTTAAGGCGAAAGATTAGAAATCTTTTGGGCTTTGCCCGCGCAGGTTCGAGTCCTGCAGTTGTCG

>chrX.trna14-AGA

GGCAACTTGGCCGAGTGGTTAAGGCGAAAGATTAGAAATCTTTTGGGCTTTGCCCGCGCAGGTTCGAGTCCTGCAGTTGTCG

>chrXII.trna21-AGA

GGCAACTTGGCCGAGTGGTTAAGGCGAAAGATTAGAAATCTTTTGGGCTTTGCCCGCGCAGGTTCGAGTCCTGCAGTTGTCG

>chrXIII.trna18-SerAGA

GGCAACTTGGCCGAGTGGTTAAGGCGAAAGATTAGAAATCTTTTGGGCTTTGCCCGCGCAGGTTCGAGTCCTGCAGTTGTCG

>chrIII.trna4-CGA

GGCACTATGGCCGAGTGGTTAAGGCGAGAGACTCGAATGGAATAAAAAGTTCGGCTATCTCTTGGGCTCTGCCCGCGCTGGTTCAAATCCTGCTGGTGTCG

>chrVI.trna7-GCT

GTCCCAGTGGCCGAGTGGTTAAGGCGATGCCCTGCTATTTCCTCAGAAAAGCAATTAGGCATTGGGTTTTACCTGCGCAGGTTCGAATCCTGTCTGTGACG

>chrXV.trna3-GCT

GTCCCAGTGGCCGAGTGGTTAAGGCGATGCCCTGCTATTTCCTCAGAAAAGCAATTAGGCATTGGGTTTTACCTGCGCAGGTTCGAATCCTGTCTGTGACG

>chrIX.trna4-TGA

GGCACTATGGCCGAGTGGTTAAGGCGACAGACTTGAAATCTGTTGGGCTCTGCCCGCGCTGGTTCAAATCCTGCTGGTGTCG

>chrV.trna17-TGA

GGCACTATGGCCGAGTGGTTAAGGCGACAGACTTGAAATCTGTTGGGCTCTGCCCGCGCTGGTTCAAATCCTGCTGGTGTCG

>chrXVI.trna3-TGA GGCACTATGGCCGAGTGGTTAAGGCGACAGACTTGAAATCTGTTGGGCTCTGCCCGCGCTGGTTCAAATCCTGCTGGTGTCG

**Figure S3.** Gene sequences of *S. cerevisiae* tRNA^Ser^ isoacceptors for cytoplasmic translation. Sequences were retrieved from the GtRNAdb (<http://gtrnadb.ucsc.edu/index.html>). Introns are included in these sequences.


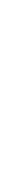

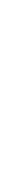

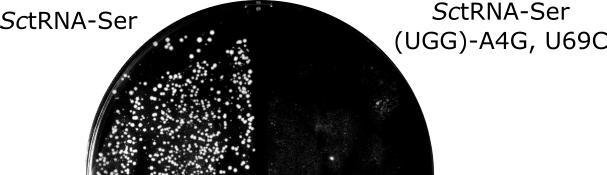


**Figure S4.** Human tRNA^Ser^_UGG_ with G4:C69 is toxic in *S. cerevisiae***.** *URA3* centromeric plasmid (YCplac33) expressing *S. cerevisiae* [*SUP17; Sc*tRNA^Ser^_UGA_] or *Hs*tRNA^Ser^_UGG_-G4:C69 were transformed into the wild-type yeast strain BY4742 and grown for 3 days at 30°.


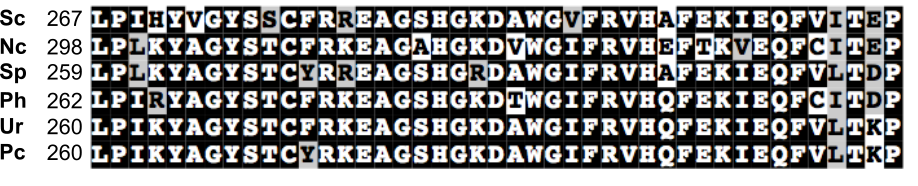


**Figure S5**. Alignment of cytoplasmic SerRS motif 2 sequences from yeast and fungal species. Motif 2 sequences from *Uncinocarpus reesii* (Ur), *Schizosaccharomyces pombe* (Sp), *Penicillium chrysogenum* (Pc), *Pseudozyma hubeiensis* (Ph), *Neurospora crassa* (Nc) and *Saccharomyces cerevisiae* (Sc) were aligned using the default settings of MUSCLE (<https://www.ebi.ac.uk/Tools/msa/muscle/>).

**
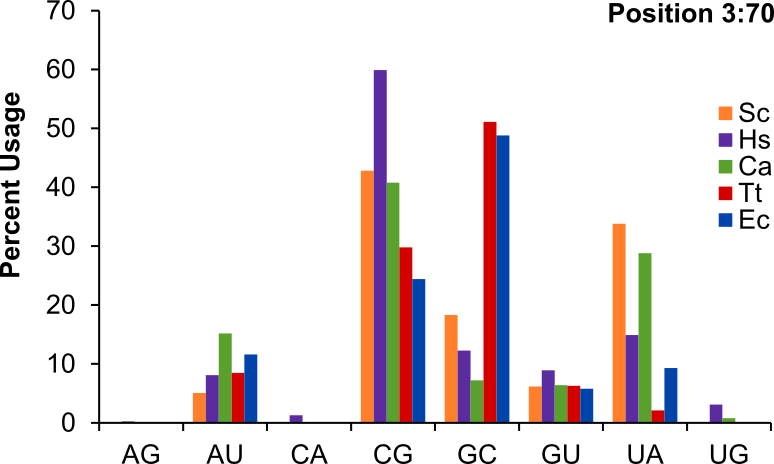
**

**Figure S6.** Percent distribution of the base pair at 3:70 in the tRNAs in *Saccharomyces cerevisiae*, *Homo sapiens*, *Candida albicans*, *Thermus thermophilus and Escherichia coli*. tRNA sequences were obtained from the GtRNAdb (<http://gtrnadb.ucsc.edu/index.html>). Likely pseudogenes were excluded.


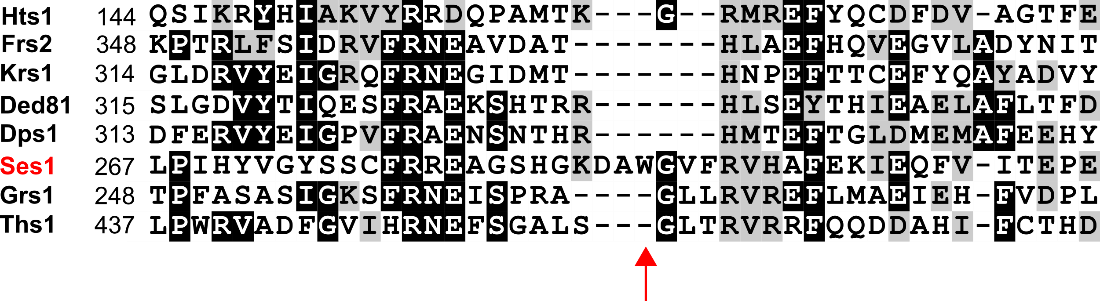


**Figure S7.** Motif 2 sequences from the yeast class II aaRS enzymes Hts1 (residues 144-182), Frs2 (348-385), Krs1 (314-351), Ded81 (315-353), Dps1 (313-351), Ses1 (SerRS, 267-310), Grs1 (248-287) and Ths1 (437-477). Alignments were performed with MUSCLE. The tryptophan of SerRS is indicted by an arrow.


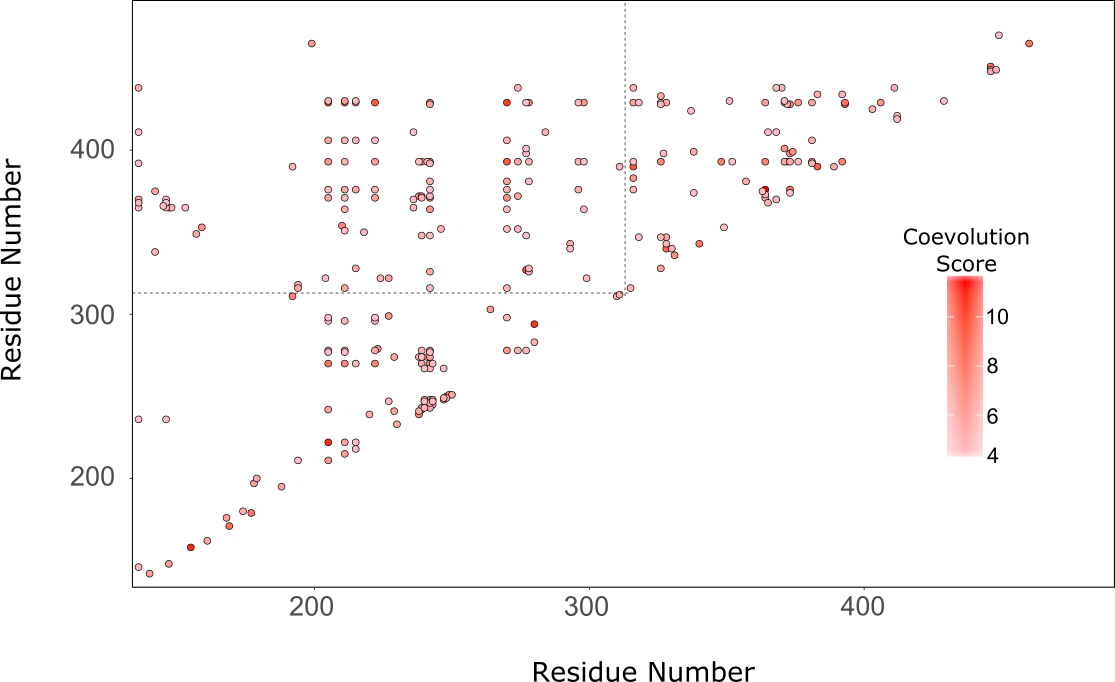


**Figure S8.** Co-evolution of cytoplasmic SerRS. Potentially coevolving residues (with coevolution scores greater than 4) were predicted from 667 SerRS sequences using MIpToolset (Dickson and Gloor 2014). Numbering corresponds to the human SerRS sequence. The N-terminal residues, corresponding to the alpha helices which bind the variable arm of tRNA-Ser were omitted due to variation in length. The dotted lines represent Arg313 (Trp290 in yeast).
